# Feasibility and early effectiveness of the Tell‐us Card communication tool to increase in‐hospital patient participation: a cluster randomised controlled pilot study

**DOI:** 10.1111/scs.12909

**Published:** 2020-09-21

**Authors:** Elise van Belle, Getty Huisman‐De Waal, Hester Vermeulen, Maud Heinen

**Affiliations:** ^1^ Radboud Institute for Health Sciences IQ Healthcare Radboud University Medical Center Nijmegen The Netherlands; ^2^ Department of Cardiology Radboud University Medical Center Nijmegen The Netherlands; ^3^ Faculty of Health and Social Studies HAN University of Applied Sciences Nijmegen The Netherlands

**Keywords:** nursing care, patient participation, communication, hospitals, quality of health care, nurse–patient relations, feasibility studies, outcome assessment (health care)

## Abstract

**Background:**

Patient participation is fundamental to nursing care and has beneficial effects on patient outcomes. However, it is not well embedded yet and little is known on how nurses could effectively stimulate patient participation in hospital care. The Tell‐us Card is a communication tool for inviting patients to talk about their preferences and needs, and to increase patient participation in daily care.

**Objectives:**

To assess feasibility and early effectiveness of the Tell‐us Card communication tool for enhanced patient participation during hospitalisation.

**Design and method:**

A pilot cluster randomised controlled study design was used including four nursing wards. Effectiveness was measured with the Individualized Care Scale (ICS) and the Quality from the Patients’ Perspective (QPP) questionnaire. Linear mixed model analysis was used for analysis. Feasibility was assessed with an evaluative questionnaire for patients and nurses and by reviewing the content of Tell‐us Cards using the Fundamentals of Care Framework (FOCF) for analysis. Ethical approval was attained.

**Results:**

Data of 265 patients showed a significant increase at one intervention ward on the ICS (effect size 0.61, p = 0.02) and most ICS subscales. No effect was visible on the QPP. The majority of patients regarded the intervention as beneficial; nurses however experienced barriers with incorporating the Tell‐us Card into daily care. Analysis of the Tell‐us Card content showed many elements of the FOCF being mentioned, with most patients indicating psychosocial needs like being involved and informed.

**Conclusions:**

This pilot study showed a positive early effect of the Tell‐us Card communication tool on patient participation, although integration in daily nursing care appeared to be complex and an optimal fit has not yet been reached. Patients were positive about the intervention and wrote meaningful issues on the Tell‐us Cards. More research is needed on how to incorporate patient participation effectively in complex hospital care.

## Introduction

Hospitalised patients’ participation in care is challenging but has various beneficial effects in patient safety (1), adherence to therapy or lifestyle advices (2), and both patient (3) and healthcare professionals work satisfaction (4). There is not one clear definition of patient participation, and the concept is interchangeably used with terms like patient‐centredness, shared decision‐making, person‐centred care, and patient empowerment or engagement. Patient participation in nursing practice is defined by Sahlsten et al. as an established relationship between nurse and patient, a surrendering of power or control by the nurse, shared information and knowledge, and active engagement together in intellectual or physical activities (5). This established relationship forms the core of effective patient participation and person‐centred care (6). Participation can be enacted at different degrees ranging from the patient being informed to being in full control and can be situated at the micro‐, meso‐ and macro‐level (7). To achieve this partnership, the patient’s view as an expert must be considered important and requires activation of both the patient as well as the healthcare provider (8).

Communication should therefore be characterised by respect, empathy and recognition of the patient as an individual as well as a partner in health care (9). Communication is also defined as a fundamental of care and foundation of any healthcare provider to patient interaction (10). Through effective communication, patients can participate in their care by setting achievable short‐ and long‐term goals to regain control over their bodily functions as well as to regain a sense of personal integrity and sense of self (11). Observational studies show that the nurses’ controlling approaches can be a hindering factor (12). Patient‐centred communication and patient participation is often lacking in during hospitalisation as there is little dialogue between patients and nurses on what patients expect or how they want to participate (5). Also, care and discharge plans often fail to take patient preferences into consideration (13). Overall patient participation in acute health care is lacking, and evidence on interventions to improve patient participation in fundamental nursing care is limited (6).

A promising tool to improve patient participation during hospitalisation is the Tell‐us Card (Tell‐us Card) (15, 16, 17). The Tell‐us Card is a postcard‐sized paper card which is handed to patients on a daily basis. Patients are invited to write down what is important to them for that day or before discharge. At a mutually agreed moment of time, the content of the card is discussed and possible follow‐up actions are planned and registered in the patient’s file. Jangland et al. tested the effectiveness of the Tell‐us Card in a population of Swedish patients admitted to a surgical nursing ward and showed significant improvements in patients’ abilities to participate in decisions about their care (17). Following the MRC framework for complex interventions, the Tell‐us Card needs to be tailored and pilot tested to explore feasibility and small‐scale effects before implementation in other settings (18). In this pilot trial, the researchers set out to (1) determine small‐scale effects of the Tell‐us Card intervention, (2) evaluate user experiences and (3) evaluate the appropriateness of outcome measures. The general aim of this study therefore was to assess feasibility and early effectiveness of the Tell‐us Card in the Dutch hospital setting.

## Methods

### Design

To assess feasibility and early effectiveness, a cluster randomised controlled study (CRTC) design was used. With this design, we aimed to compare effects within and between clusters. As nursing care can differ between surgical and internal specialties, two surgical wards and two cardiology wards were included. Both surgical wards resided within the same university hospital, as well as one of the cardiology wards. The other cardiology ward (intervention group) was located in a nearby regional hospital. The wards were assigned to either control or intervention by a random draw by an independent researcher. Assessments were conducted at baseline (T0) and 3 months after the start of the intervention (T1). The CONSORT statement extension for randomised pilot and feasibility trials (19) was used for reporting.

### Participants

All adult patients (age > 18) with an expected hospital stay of at least one day and a diagnose fitting the wards specialism were included. Patients were excluded if they were not able to speak or write in Dutch, had mental impairments, or were not willing or able to give informed consent. The surgical cluster consisted of a head and neck surgical ward and a ward for neurosurgical and plastic surgery. The cardiology wards both admitted patients with acute and chronic cardiac conditions. At T0 patient characteristics were compared to determine comparability. Nurses working on the wards were vocational of bachelor educated, and had a nurse‐to‐patient ratio of 1:4 during the day and 1:6–8 during the evenings.

### The Tell‐us Card intervention

The Tell‐us Card is a communication tool to elicit what patients regard as important at that moment or before discharge (Box 1). The control group received care as usual. Permission to use the Tell‐us Card was obtained from the original researcher (17). The card was translated to Dutch by the authors and slightly modified based on input from the wards’ nurses (20).

Box 1 **Tell‐us Card protocol**

The nurse…

1. Gives the double‐sided Tell‐us Card once a day to each patient.

Side A: ‘Tell us! We want to involve you in your care as much as possible. What is important to you today or before discharge? What are your needs, or what information do you want? What do you want us to know about you as a person? Are there arrangements that need to be taken care of? What things can you do yourself, and where do you need help with? We would like to invite you to write down your questions, wishes, worries and ideas on the back of this card. The nurse who takes care of you will discuss these with you’.

Side B: ‘Tell us! Write down on this card what is important to you. Your nurse will discuss this with you”. Followed by: "This is important for me:*…*

…………………………………………………………………………………………

…………………………………………………………………………………………’

- Goes back to the patient after an agreed amount of time to discuss the card and to talk about what is important.
- Establishes with the patient if/what follow‐up actions are needed and by whom.
- Reports the findings and agreed upon actions in the patient’s file.
- Reports back to the patient if/what follow‐up actions are undertaken.

### Preparing for implementation

In line with the MRC framework (21), the implementation of the Tell‐us Card was systematically tailored using the Intervention Mapping framework (22). As described in (20) van Belle et al. 2018, focus group meetings were conducted after T0 assessment to identify the nurses’ knowledge, skills, attitude, self‐efficacy and outcome expectations regarding the intervention, which was used in the training. Nurses were trained by means of an e‐learning and group discussion. At both wards, a core group of nurses was formed to guide implementation by stimulating the use of the Tell‐us Card, addressing questions from the nurses and providing feedback. Additional strategies during the intervention period included educational and feedback visits to the wards’ nurses where the study procedures were repeated, questions from nurses answered and progress on received questionnaires was shared (20). The patient questionnaires were piloted with four patients and deemed understandable and acceptable in length.

### Study procedures

All included patients received written and verbal information about the study and signed an informed consent. At T0 and T1, patients at the intervention and control wards fitting the inclusion criteria received a questionnaire with a prepaid return envelope to be filled in at home after discharge. Nurses were trained to ask patients’ consent to participate and were responsible for handing out the questionnaire upon discharge. All activities aimed at the nurses, such as focus groups and training, started after T0 assessments. The filled in Tell‐us Cards were stored in a closed container in the nurses’ station.

### Primary outcomes

Effectiveness was assessed by a patient questionnaire at T0 and T1 including demographic information, the Individualized Care Scale (ICS) (23) and the Short form Quality of the Patients Perspective questionnaire (24). The Short form Quality from the Patients Perspective (QPP) (24) questionnaire is an 18‐item scale and measures four dimensions of care: medical–technical competence (four items), an identity‐orientation approach (10 items), physical–technical conditions (three items) and socio‐cultural atmosphere (five items) (Table 3). Items were rated on a scale of 1 (‘do not agree at all’) to 4 (‘completely agree’); additionally, each item had a ‘not applicable’ response alternative. It was chosen to compare results with the Swedish Tell‐us Card study (17). Translation from English to the Dutch language was conducted by two Dutch researchers and a certified translator using a forward–back translation (20). The ICS (23, 25) is a 34‐item scale assessing the individualised care experience. The scale is divided into two parts of 17 questions each: (A) the practice of individualised care during nursing interventions and (B) the perception of individuality in care. Both parts include three domains: the clinical situation (seven items), the personal life situation (four items) and decisional control over care (six items) (Table 2). See Appendices S1 and S2 for the abbreviated questions and item scores on the QPP and ICS. Each item is rated on a 5‐point scale of 1, fully disagree, to 5, fully agree with the statement. The scale has a neutral midpoint and has been validated for the Dutch healthcare context (26). Results of the questionnaires were examined on missings and distribution to evaluate usefulness and feasibility.

Feasibility was assessed by examining the content of the Tell‐us Cards and asking nurses and patients by means of a questionnaire about their experiences with the Tell‐us Card. In this questionnaire, they were asked to indicate how often they used the Tell‐us Card, if they were properly instructed, if they perceived the card as helpful and to what extent they appreciated the use of the Tell‐us Card. With each question, there was the opportunity to add remarks.

### Sample size

To assess effectiveness in a small‐scale pilot study without a predetermined level of precision it is advised to have a sample size of 24–30 patients to get a reliable estimation on the effect of the intervention (27). This study set out to include 35 patients at each ward at T0 and T1.

### Analysis

SPSS (28) was used for the quantitative analysis. Means, standard deviations, ranges and percentages were used to describe the data, and t test and chi‐square analysis were used to calculate differences between wards at T0. Because of the hierarchical structure of this study (patients nested within wards), the analyses were based on a linear mixed‐effect model for the ICS and QPP outcomes. Reported differences are changes in score between T1 and T0 and between intervention and control wards. Statistical significance in all tests was assumed at the 0.05 level, based on two‐sided tests. Reported effect size signifies the change on the 4‐ or 5‐point scale.

The content of the Tell‐us Card was analysed by using framework analysis and thematic analysis (29).

Data were categorised according to the Fundamentals of Care Framework (30) as this gives a full overview of physical, psychosocial and relational needs. Additional thematic analysis was used for results not fitting the framework. Coding was done independently by two researchers (EvB and MH); afterwards, codes were compared and differences discussed until consensus was reached. The open questions in the questionnaire were analysed using independent open coding (EvB and MH), following axial coding and the identification of themes (29).

### Ethical considerations

The study was approved by the regional Ethical Review Board (approval number 2014‐1350) and the participating ward’s management. According to the Dutch national legislation and as judged by the local Medical Ethics Committee, the study is noninvasive and does not fall under the scope of the Medical Research Involving Humans Subjects Act (31). Patients and nurses were informed about the right to decline from participation without giving any reason at any time. All data were analysed anonymously, with to persons retraceable information stored separately from the data.

## Results

The study took place between November 2014 and July 2016. Quantitative baseline data were gathered on four wards in a 6‐month period from December 2014 to May 2015. The intervention period started on both wards in October 2015 and lasted 3 months. The data at T1 were gathered between December 2015 and July 2016, with a mean duration of 3.5 months.

### Participant flow and recruitment

**Figure 1:**
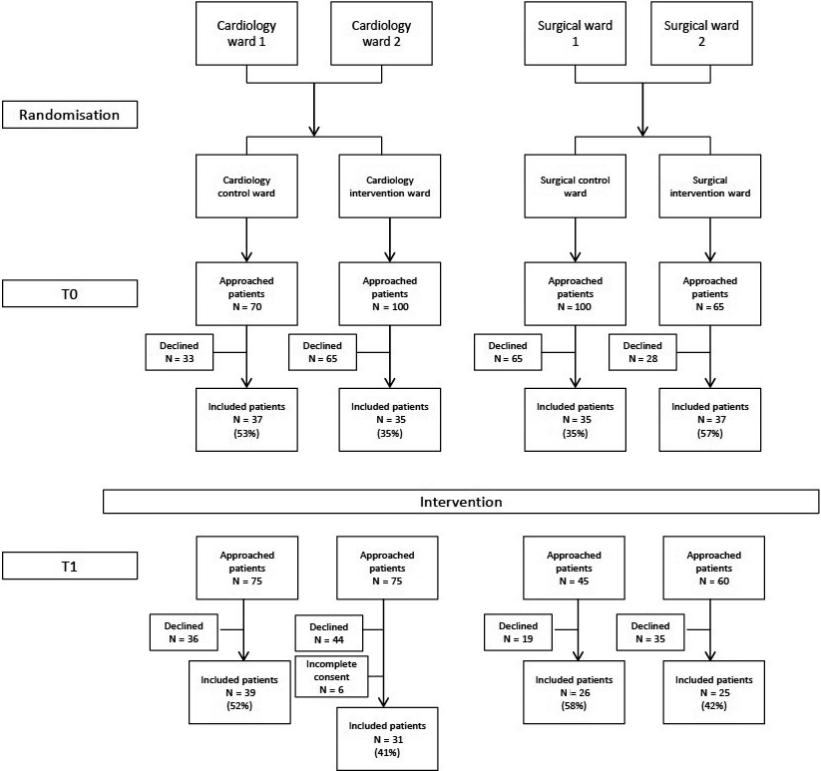
Flow of patients through the study.

Twenty per cent of the patients at the surgical ward and 10% of the patients at the cardiology ward did not meet the inclusion criteria. A total of 265 patients completed the questionnaire, with 144 patients at T0 and 121 patients at T1 (Figure 1). The response rate varied between 35% and 57% at T0, and between 41% and 58% at T1. At the surgical intervention ward, 14 of the 20 nurses (70%) filled in the evaluative questionnaire; at the cardiology intervention ward, this was 42 out of the 60 nurses (70%). The Tell‐us Card was handed out 158 times to 107 individual patients; 123 times to 72 patients (mean 1.7 per patient) at the surgical ward, and 41 times to 35 patients (mean 1.2) on the cardiology ward. In total, 108 cards (70%) were filled in by patients.

The T0 measurements were completed within the predetermined 3‐month period. At T1, both cardiology wards met the patient sample of 35 within this time period, with the intervention ward including 37 patients. However due to incomplete informed consent forms, six questionnaires needed to be excluded from analysis, resulting in 31 included patients. The surgical cluster experienced difficulties in following up the study protocol with regard to informed consent procedures and handing out questionnaires at discharge. Therefore, the data collection period at the surgical cluster was extended to 5 months, after which 25 and 26 patients at, respectively, the intervention and control ward had returned the questionnaire. Patients at the surgical intervention ward were not able to assess feasibility of the Tell‐us Card because the intervention period ended before the start of T1 assessments. As it was hypothesised that the intervention period enhanced patient participation within the care process, it was decided to conduct effectiveness analysis as planned.

### Patient characteristics

t Tests and chi‐square tests showed no significant difference at T0 of patient characteristics within the clusters with respect to age, gender, educational level and length of admission (Table 1). On average, 63% of the respondents at the cardiac wards were male, and 40% at the surgical wards. At T1, only gender differed significantly (p = 0.007) at the cardiology ward, with more men being included in the intervention ward. The study participants age ranged from 24 to 90 years (mean 67, SD 11.3) at the cardiology, and from 20 to 88 years (mean 54, SD 15.0) at the surgical wards.

**Table 1:** Patient characteristics

	Cardiology						Surgical					
	T0			T1			T0			T1		
	Control n = 37	Intervention n = 35	p Value *	Control n = 39	Intervention n = 31	p Value *	Control n = 37	Intervention n = 35	p Value*	Control n = 26	Intervention n = 25	p Value*
Gender			0.62			0.007			0.13			0.41
Male (%)	27 (73)	23 (66)		16 (41)	23 (72)		10 (29)	17 (46)		10 (38)	12 (50)	
Age			0.86			0.96			0.33			0.08
Median age (SD) years	67.7 (12.8)	65.8 (9.4)		66.9 (11.8)	68.5 (11.0)		52 (13.4)	58.6 (18.4)		51.7 (15.1)	63.8 (12.4)	
Level of education^a^			1.00			0.11			0.73			0.65
Primary education (%)	12 (32)	11 (31)		16 (43)	9 (31)		9 (24)	7 (20)		5 (19)	6 (24)	
Secondary education^b^ (%)	12 (32)	11 (31)		11 (28)	17 (59)		15 (41)	17 (48)		10 (38)	13 (52)	
Bachelor degree or higher (%)	13 (32)	12 (35)		10 (26)	3 (10)		13 (35)	9 (26)		11 (42)	6 (24)	
Length of admission			0.62			0.49			0.82			0.33
1–2 days (%)	0 (0)	1 (3)		3 (8)	1 (3)		10 (28)	10 (27)		15 (59)	3 (12)	
3–4 days (%)	8 (22)	8 (23)		12 (31)	4 (13)		6 (17)	6 (16)		3 (11)	4 (16)	
5–6 days (%)	12 (32)	11 (32)		3 (8)	7 (23)		8 (23)	8 (22)		3 (11)	4 (16)	
7–9 days (%)	4 (11)	6 (17)		8 (20)	8 (26)		9 (26)	7 (19)		3 (11)	6 (24)	
10 or more days (%)	10 (30)	7 (20)		11 (30)	8 (26)		2 (6)	5 (14)		2 (8)	8 (32)	

* p Value were based on chi‐square test for categorical variables and t test for continuous variables.

^a^ Level of education is defined following the ISCED 2011 (48).

^b^ Secondary education and postsecondary, nontertiary education.

#### Primary outcomes and estimation

##### Individualized Care Scale

The linear mixed‐effect model analysis for the cardiology patients shows a significant improvement at T1 on 6 out of 9 (sub)scales (Table 2). This effect is established at different levels; the total ICS scale (effect size 0.61, p = 0.02), both part A (ES: 0.62, p = 0.04) and part B (ES: 0.73, p = 0.004), and subscales ICS‐A Personal life situation (ES: 0.89, p = 0.03), ICS‐B Personal life situation (ES: 1.07, p = 0.002) and ICS‐B Decisional control over care (ES: 0.57, p = 0.01). Results in the surgical cluster showed no significant differences between T0 and T1. In both control wards, there appears to be a declining trend over time, as five out of six subscales score lower on T1 at the surgery wards, and four out of six at the cardiology ward.

**Table 2:** Individualized Care Scale

	Cardiology				Effect^a^	p	Surgical				Effect^a^	p
	Control		Intervention				Control		Intervention			
	Mean T0 (SD)	Mean T1 (SD)	Mean T0 (SD)	Mean T1 (SD)			Mean T0 (SD)	Mean T1 (SD)	Mean T0 (SD)	Mean T1 (SD)		
	n = 37	n = 39	n = 35	n = 31	n = 142		n = 35	n = 26	n = 37	n = 25	n = 123	
Individualized Care Scale	4.19 (0.66)	3.93 (0.87)	3.86 (0.86)	4.21 (0.57)	0.61	0.02	4.20 (0.57)	3.90 (0.82)	4.01 (0.96)	3.93 (0.77)	0.22	0.45
ICS‐A	4.06 (0.77)	3.71 (1.01)	4.03 (0.92)	4.06 (0.63)	0.62	0.04	4.10 (0.66)	3.74 (1.01)	3.88 (0.97)	3.81 (0.87)	0.29	0.38
ICS‐A Clinical	4.26 (0.83)	4.09 (1.01)	4.03 (0.94)	4.14 (0.83)	0.28	0.37	4.26 (0.62)	3.82 (0.89)	4.04 (0.93)	4.03 (0.94)	0.43	0.17
ICS‐A Personal	3.49 (1.15)	3.75 (1.36)	3.14 (1.22)	3.75 (0.89)	0.89	0.03	3.49 (1.15)	3.35 (1.43)	3.65 (1.24)	3.43 (1.07)	0.01	0.98
ICS‐A Decisional	4.20 (0.74)	4.22 (1.05)	3.95 (0.91)	4.22 (0.71)	0.58	0.05	4.26 (0.71)	3.94 (1.06)	3.98 (1.10)	3.80 (0.93)	0.14	0.69
ICS‐B	4.30 (0.60)	4.01 (0.78)	3.92 (0.89)	4.37 (0.57)	0.73	0.004	4.28 (0.62)	4.06 (0.80)	4.04 (0.97)	4.06 (0.82)	0.24	0.43
ICS‐B Clinical	4.30 (0.72)	4.25 (0.89)	3.96 (1.00)	4.25 (0.72)	0.52	0.07	4.35 (0.64)	3.93 (1.04)	4.12 (1.05)	4.04 (0.98)	0.34	0.32
ICS‐B Personal	3.94 (0.91)	3.62 (1.10)	3.33 (1.12)	4.08 (0.84)	1.07	0.002	3.87 (1.01)	3.66 (1.08)	3.70 (1.08)	3.55 (0.90)	0.07	0.86
ICS‐B Decisional	4.61 (0.47)	4.66 (0.78)	4.03 (0.70)	4.66 (0.48)	0.57	0.01	4.44 (0.64)	4.44 (0.53)	4.31 (0.91)	4.41 (0.73)	0.11	0.70

^a^ Reported differences are changes in score between T0 and T1 and between control and intervention wards.

The scores of the four wards show quite similar patterns. Looking at the results of the ICS, only 22% of the items had a mean score below 4.00 on the 5‐point scale, with a lowest mean score of 3.14 (Appendix S1). The highest scoring subscale (on all wards at both T0 and T1) concerned the nurse inviting the patient to be involved in his or her care (ICS‐B decisional control over care), with mean scores ranging from 4.03 to 4.66. This is mainly due to the questions concerning the items ‘patients’ ability to follow instructions received in the hospital’ (range 4.66–4.97) and ‘patients making their own decision on when to wash’ (range 4.34–4.87). The questions in this subscale (ICS‐B Decisional) relating to ‘patients’ expressed wishes have been considered in care’ (range 4.03–4.60) and ‘patients taking part in decision‐making’ (range 3.87–4.43) scored lower. The two lowest scoring subscales on all wards concerned incorporating the patient’s personal life situation into the hospital care (both subscale A and B), with average scores of 3.51 for subscale A and 3.72 for subscale B. The question ‘nurses asking about previous experiences of hospitalisation’ (range 2.59–3.58) scored either lowest or in the bottom 3 on all wards.

##### Quality of the Patient’s Perspective

The results on the QPP showed no significant change at any of the wards at T1. Also, approximately 75% of all mean scores ranged between 3.50 and 4.00 (Table 3), meaning that patients scored high on the questionnaire’s 4‐point scale at T0 and T1. The highest scoring question on all wards concerned ‘the patient’s friends and family being treated well’ (range 3.80–4.00) (Appendix S2). The lowest scoring questions were about ‘whether the care was determined by the patient’s requests and needs, rather than staff procedures’ (range 3.13–3.63) and ‘talking to the doctor in private when the patient wanted’ (range 2.71–3.63). The latter was answered by 43% of patients as being ‘not applicable’ (NA), making it one of the three items with highest NA rates. Others were ‘being able to talk to a nurse in private’ (35% NA) and ‘having access to necessary care equipment’ (35% NA). As patients were asked to fill in two questionnaires, the data showed no signs of fatigue or inconsistencies, or higher numbers of questions that were not answered.

**Table 3:** Quality from the patient’s perspective

	Cardiology				Effect^a^	p	Surgical				Effect^a^	p
	Control		Intervention				Control		Intervention			
	Mean T0 (SD)	Mean T1 (SD)	Mean T0 (SD)	Mean T1 (SD)			Mean T0 (SD)	Mean T1 (SD)	Mean T0 (SD)	Mean T1 (SD)		
	n = 36	n = 34	n = 35	N = 31	n = 136		N = 35	n = 26	n = 37	n = 25	n = 123	
Quality from the Patient’s Perspective	3.74 (0.33)	3.78 (0.68)	3.61 (0.36)	3.64 (0.58)	−0.02	0.91	3.81 (0.25)	3.72 (0.24)	3.68 (0.46)	3.76 (0.27)	0.06	0.56
Medical–technical competence	3.79 (0.41)	3.78 (0.49)	3.72 (0.44)	3.63 (0.63)	−0.08	0.64	3.84 (0.31)	3.82 (0.26)	3.71 (0.51)	3.89 (0.24)	0.05	0.64
Identity‐oriented approach	3.77 (0.38)	3.70 (0.48)	3.60 (0.49)	3.65 (0.61)	0.12	0.50	3.93 (0.30)	3.70 (0.37)	3.68 (0.56)	3.77 (0.31)	0.04	0.79
Physical–technical conditions	3.61 (0.48)	3.63 (0.55)	3.59 (0.47)	3.63 (0.62)	0.02	0.90	3.82 (0.35)	3.74 (0.47)	3.66 (0.51)	3.73 (0.48)	0.08	0.52
Social cultural atmosphere	3.72 (0.43)	3.61 (0.58)	3.56 (0.52)	3.63 (0.60)	0.18	0.34	3.73 (0.37)	3.63 (0.35)	3.67 (0.52)	3.65 (0.49)	0.13	0.30

^a^ Reported differences are changes in score between T0 and T1 and between control and intervention wards.

##### Feasibility

The evaluative questions were answered by 31 cardiology patients. Most patients (78%) received the Tell‐us Card once. Patients indicated that the aim of the card was clear (96%). About three quarters of the patients (74%) indicated the card had helped ‘somewhat’ or ‘very much’ to tell the nurse what was important to them. Patients responded to the open question ‘What do you think of the Tell‐us Card?’ that it helped them raise issues, they saw it as a tool to improve the quality of care, and that they used the card as a means to write down their experiences or questions. Some patients indicated that they preferred not to use the card and just talk to the nurses.

All nurses on the surgical ward and 73% of the nurses on the cardiology ward felt they had been well‐instructed on how to use the Tell‐us Card. At both wards, about two‐thirds of the nurses (62%–64%) indicated that they had used the Tell‐us Card one to three times during the intervention period. About one‐third of the nurses (31%–29%) on both wards stated to have used it more than five times. A majority of the nurses (82% at cardiology and 62% at the surgical ward) indicated that they did not think that the Tell‐us Card really helped patients to express what was important to them. Main barriers for nurses were that they felt it had little additional value, and patients not knowing what to write down. Nurses also stated to expect their patients to speak up and to prefer face‐to‐face conversation instead of a card, which indicates regarding the card as a substitute for conversation instead of a tool to initiate conversation. Additionally, registering the content and follow‐up of the Tell‐us Card in the patients’ file and handing out questionnaires at discharge were regarded as administrative burden.

##### Tell‐us Card content

The content of the 108 Tell‐us Cards was coded based on the physical, psychosocial and relational elements of the Fundamentals of Care Framework (FoCF). Many cards raised more than one topic, such as a cardiology patient writing ‘Important for me is to empathize, that they listen to me, give me the right advice, and give me genuine attention’. In this example, three elements were coded (empathy, active listening and being involved and informed). Framework analysis leads to the identification of 149 individual codes connected to 24 of the 29 fundamentals of care (Table 4). Two topics, a hygienic environment and being satisfied about care, were not part of the FoCF.

**Table 4:** Tell‐us Cards themes

Physical elements	No. of cards	Psychosocial elements	No. of cards	Relational elements	No. of cards
Personal cleansing	2	Communication	2	Active listening	2
Toileting needs	1	Being involved and informed	29	Empathy	1
Eating and drinking	5	Privacy	1	Engaging with patients	6
Rest and sleep	5	Dignity	0	Compassion	6
Mobility	3	Respect	0	Being present	1
Comfort	10	Education and information	0	Supporting and involving families and carers	1
Safety	4	Emotional well‐being	1	Helping patients to cope	5
Medication management	4	Choice	1	Working with patients to set, achieve, and evaluate progression of goals	3
Hygienic environment^a^	5	Having values and beliefs considered and respected	0	Helping patients to stay calm	7
		Social engagement, company, and support	1		
		Feeling able to express opinions and needs without care being compromised	0		
		Having interests and priorities considered and accommodated	24		
		Being satisfied about care^a^	19		

^a^ Is not part of FoC framework.

Most cards related to the psychosocial elements of the FoCF, with ‘being involved and informed’, ‘having interests and priorities considered and respected’, and ‘being satisfied about care’ being used in 78 of the 149 identified issues. Many patients want to be informed about medical treatment and results from examinations. A cardiology patient: ‘Talk to me when the medication is changed. Why they change it and information on what I am taking them for. This is not always happening’. Patients wanted nurses to inform them about self‐care at home or at the hospital, explain their actions during care and let them know what the day was going to be like. All physical elements of the FoCF were identified. Most were about eating and drinking, rest and sleep, and comfort. A surgical patient stated ‘I feel really bad. Did not sleep last night. Despite pain medication my pain did not significantly decrease. I have cold sweats, I am nauseous, my stomach hurts and I feel weak. I want to go home, but only if I get sufficient pain medication’. Also, all nine relational elements were identified, with patients wanting the nurses and other healthcare professionals to be friendly, respectful, involved and to pay attention to them as a person. A patient on the cardiology ward wrote ‘I want a personal conversation which shows that the nurse understands me. Sharing laughter and tears, a pat on the back, holding your hand. Being there for the patient who has been in an emotional rollercoaster since being admitted’. Some patients wrote that they were anxious or fearful about pain, examinations, or anything happening to them and needed help coping or staying calm. A surgical patient responded ‘I am afraid of choking, I want to be sure this won’t happen and to have help with this at home’.

## Discussion

The results showed a significant impact of the Tell‐us Card intervention on most (sub)scales of the ICS at one intervention ward. Patients were most satisfied with the domain of decisional control. Incorporating the patient’s personal life into care and determining care based on patients’ needs scored the lowest. There was no significant effect on the Quality from the Patient’s Perspective questionnaire (QPP). Patients valued the Tell‐us Card and wrote down a variety of topics. Nurses experienced difficulties in using the Tell‐us Card communication tool despite their training and involvement in tailoring the intervention to their wards.

The topics on the Tell‐us Cards reflected most of the elements of patient participation (5), as patients stressed the importance of good relationships with nurses, they wanted to be informed, to express their wishes and needs regarding discharge or home care, and they wanted to share their worries. Also the core of the Fundamentals of Care Framework (32) is related to these outcomes, which describes a positive professional relationship being based on trust, focus, knowing, anticipation and evaluation. A trusting relationship is regarded as essential in identifying patients’ needs, and necessary for nurses to be responsive and attentive to changes in a patients’ health condition (32). Difficulties experienced by the nurses in this study underlined the unfamiliarity with patient participation in acute health care (6). Although a patient‐centred approach is stressed at the (inter)national level and is recognised to have a significant impact on patient outcomes (33, 34), applying it in daily practice remains challenging. Nurses emphasised their lack of time and the patient’s unfamiliarity with being an active participant in care as problematic, which is in line with barriers identified in previous research on patient participation (12, 35, 36).

The Tell‐us Card might not be regarded as the most appropriate tool for enhanced patient participation, as nurses indicated the tool to redundant and experienced difficulties in incorporating the intervention in daily care; patients however valued the card and addressed important topics. Literature shows that nurses in general feel confident about their communication skills in promoting patient participation (37, 38) but also that staff communication is often perceived as disconnected and inadequate (39, 40, 41) with nurses limiting or even avoiding communication (12, 42). Literature also shows that healthcare professionals and patients mainly understand patient participation as giving or receiving information (43), and patients often perceive an imbalance in power (42, 44). In addition, most of today’s nursing education insufficiently incorporates how to address patient participation adequately in daily care (45). This requires from nurses to take the lead in enhancing patient participation in their care, as the patient’s confidence to participate will diminish when nurses display behaviours that are unsupportive of patient participation (41).

In the literature several factors are identified as enabling. Tobiano et al. advised informing patients about their role in care, and making the care process predictable for the patient while leaving room for tailored participation levels (35). Involving patients in care planning and discussing long and short‐term goals as well as discussing process conflicting expectations and roles (41, 46) are regarded as beneficial to proactively empower patients to participate (8, 39, 47). Evidence suggests that nurses in strategic leadership positions as well as ward or hospital management advocating the need for patient‐centred care and participation are necessary to really make a change towards a more patient‐centred care (48). Thus, without participation‐focused leadership and a clear vision on how patient participation should be enacted, the adequate use of a tool for enhanced patient participation such as the Tell‐us Card will remain difficult.

Lastly, the appropriateness of the measurement instruments needs to be discussed. QPP results were skewed, with only 5% of the mean scores lower than 3.00 on the 4‐point scale, and 75% above 3.50. This means that patients were already very satisfied with the items, leaving very little room for a significant change in small samples. It may therefore not be useful in detecting change in patient participation level. Additionally, several questions were regarded as not applicable by a high number of patients. Janglands’ Swedish study of the Tell‐us Card (17) did find significant results on this scale and reported lower values of ‘not applicable’. This might be due to cultural differences between the two countries. Although the results on the ICS were also positively skewed, in line with the Finnish validation study (25), a significant change was detected regardless of the small sample. The ICS therefore seems appropriate for measuring patient participation in nursing care in the Dutch hospital setting.

### Strength and limitations

A strength of this study is the cluster randomised controlled design, enabling the researchers to assess effectiveness in a complex environment and test the adequacy of the measurement instruments. Also, the developmental process preceding this pilot provided a solid base. Nevertheless, some limitations need to be mentioned. A first limitation that needs to discussed is the fact that patients of the surgical intervention ward included at the T1 assessment did not receive a Tell‐us Card. As the Tell‐us Card was handed out 123 times during the 3‐month intervention period before T1 assessments, the intervention was expected to enhance the nurses’ behaviour regarding patient participation. T1 assessments on perceived patient participation at the surgical wards were therefore carried out as planned. The results showed no significant improvement on the ICS scale, as opposed to the cardiology intervention ward. This finding however might be further strengthening the indicated effect of the actual use of the Tell‐us Card communication tool. A second limitation of the study lies in the fact that there might have been some selection bias due to nurses choosing patients they felt were more suitable or receptive for the use of the Tell‐us Card instead of giving it to all patients. Follow‐up research could benefit from assessing whether and how patient characteristics relate to a need or ability to participate in care and how nurses act upon with these differences. Third, Flottorp et al. stressed the importance of considering various influencing factors before implementation (49). This study mainly focused on issues related to the intervention itself, as well as the individual nurse and patient factors. Future research might benefit from incorporating also other, external influences related to implementation like the capacity for organisational change, including clinical nurse leadership and management.

## Conclusion

The Tell‐us Card intervention was aimed at one of the most fundamental care elements in nursing; communicating effectively with patients about their individual needs and abilities. This pilot study showed a positive early effect of the Tell‐us Card communication tool on patient participation, although integration in daily nursing care appeared to be complex and an optimal fit has not yet been reached. Patients were positive about the intervention and wrote meaningful issues on the Tell‐us Cards. More research is needed on how to incorporate patient participation effectively in complex hospital care.

## Funding

The Netherlands Organisation for Health Research and Development (ZonMw) funded the project. Project number: 520002003.

## Supporting information

**Appendix S1.** Mean scores per item Individual Care Scale.

**Appendix S2.** Mean scores per item Quality from the Patients Perspective questionnaire.
