# Supplementary material for: Feasibility and early effectiveness of the Tell‐us Card communication tool to increase in‐hospital patient participation: a cluster randomised controlled pilot study
Source: Scand J Caring Sci. 2020 Sep 21;35(3):911–22. doi: 10.1111/scs.12909 (PMC8451905; doi:10.1111/scs.12909)
Supplement: Supplementary file 1 — Appendix S1. Mean scores per item Individual Care Scale. [file SCS-35-911-s001.docx]

| Appendix S1 Mean scores per item Individual Care Scale | | | | | | | | |  | | | | | | | |
| --- | --- | --- | --- | --- | --- | --- | --- | --- | --- | --- | --- | --- | --- | --- | --- | --- |
|  | Cardiology | | | | | | | | Surgical | | | | | | | |
|  |  | | | | | | | |  | | | | | | | |
|  | Control | | | | Intervention | | | | Control | | | | Intervention | | | |
| ICS A | | ICS B | | ICS A | | ICS B | | ICS A | | ICS B | | ICS A | | ICS B | |  |
| Mean (SD)  Abbreviated question | T0 | T1 | T0 | T1 | T0 | T1 | T0 | T1 | T0 | T1 | T0 | T1 | T0 | T1 | T0 | T1 |
| 1. Feelings about illness/health  condition | 4.41 (1.14) | 4.22 (1.25) | 4.19 (1.00) | 4.08 (1.18) | 4.09 (1.12) | 4.30 (1.02) | 4.03 (1.16) | 4.26 (0.97) | 4.24 (1.08) | 3.64 (1.38) | 4.11 (1.13) | 3.58 (1.50) | 4.22 (1.25) | 4.08 (1.19) | 3.97 (1.36) | 4.12 (1.17) |
| 2. Needs that require care and  attention | 4.49 (0.99) | 4.41 (1.09) | 4.51 (0.65) | 4.25 (0.88) | 4.32 (0.95) | 4.43 (0.97) | 3.88 (1.25) | 4.35 (0.84) | 4.62 (0.70) | 4.44 (0.87) | 4.60 (0.74) | 4.23 (1.11) | 4.49 (1.04) | 4.32 (1.15) | 4.25 (1.27) | 4.28 (1.10) |
| 3. Chance to take responsibility as  far as possible | 4.62 (0.86) | 4.58 (0.94) | 4.50 (0.61) | 4.56 (0.70) | 4.53 (0.86) | 4.40 (1.00) | 4.38 (1.01) | 4.47 (0.73) | 4.76 (0.50) | 4.31 (1.23) | 4.66 (0.68) | 4.42 (1.07) | 4.46 (1.09) | 4.44 (0.96) | 4.39 (0.96) | 4.32 (0.95) |
| 4. Identify changes in how they have  felt | 4.30 (0.85) | 4.14 (1.07) | 4.14 (0.95) | 3.86 (1.22) | 4.30 (0.85) | 4.28 (1.03) | 4.03 (1.21) | 4.55 (0.69) | 4.47 (0.79) | 4.00 (1.02) | 4.54 (0.70) | 3.88 (1.18) | 4.08 (1.20) | 3.88 (1.13) | 4.31 (1.18) | 4.04 (1.17) |
| 5. Talk with patients about fears and  anxieties | 4.11 (1.27) | 3.89 (1.37) | 4.22 (0.94) | 3.94 (1.09) | 3.67 (1.36) | 3.69 (1.23) | 3.91 (1.28) | 3.97 (1.20) | 4.09 (0.93) | 3.69 (1.19) | 4.36 (0.78) | 4.00 (1.20) | 4.05 (1.10) | 4.04 (1.17) | 4.14 (1.19) | 3.88 (1.36) |
| 6. Find out how their health  conditions affect them | 4.00 (1.13) | 3.56 (1.34) | 4.19 (0.94) | 3.75 (1.18) | 3.50 (1.31) | 3.87 (0.97) | 3.85 (1.15) | 3.97 (1.07) | 3.82 (1.06) | 3.35 (1.47) | 4.27 (0.88) | 3.80 (1.32) | 3.46 (1.33) | 3.64 (1.32) | 3.76 (1.21) | 3.76 (1.30) |
| 7. What the illness/health condition  means to them | 3.89 (1.24) | 3.69 (1.39) | 4.28 (1.09) | 3.83 (1.30) | 3.82 (1.33) | 3.97 (1.00) | 3.79 (1.29) | 4.30 (0.95) | 3.85 (1.08) | 3.38 (1.44) | 3.91 (1.13) | 3.64 (1.41) | 3.54 (1.35) | 3.80 (1.25) | 3.77 (1.40) | 3.88 (1.27) |
| 8. What kinds of things they do in  their everyday life | 3.76 (1.36) | 3.58 (1.61) | 3.84 (1.09) | 3.30 (1.19) | 3.45 (1.58) | 3.77 (1.15) | 3.09 (1.40) | 3.77 (1.15) | 3.76 (1.42) | 3.52 (1.74) | 3.53 (1.24) | 3.40 (1.50) | 3.78 (1.46) | 3.32 (1.25) | 3.21 (1..27) | 3.00 (1.29) |
| 9. Previous experiences of  hospitalization | 3.16 (1.54) | 2.59 (1.50) | 3.50 (1.32) | 3.21 (1.43) | 2.88 (1.36) | 3.48 (1.26) | 2.85 (1.33) | 3.80 (1.10) | 3.58 (1.50) | 3.12 (1.56) | 3.50 (1.29) | 3.25 (1.26) | 3.53 (1.54) | 3.29 (1.27) | 3.56 (1.19) | 3.29 (1.27) |
| 10. Everyday habits | 3.70 (1.47) | 3.09 (1.58) | 4.05 (1.15) | 3.42 (1.48) | 3.00 (1.30) | 3.74 (1.13) | 3.44 (1.40) | 4.13 (1.01) | 3.50 (1.46) | 3.65 (1.44) | 3.94 (1.13) | 3.73 (1.40) | 3.65 (1.38) | 3.40 (1.26) | 3.85 (1.33) | 3.48 (1.23) |
| 11. Family to take part in their care | 3.32 (1.36) | 3.29 (1.66) | 4.32 (1.11) | 4.21 (1.18) | 3.25 (1.50) | 4.03 (1.10) | 3.84 (1.32) | 4.58 (0.77) | 3.45 (1.39) | 3.12 (1.66) | 4.41 (0.82) | 4.16 (1.07) | 3.60 (1.50) | 3.64 (1.47) | 4.11 (1.24) | 4.40 (0.91) |
| 12. Instructions to patients | 4.69 (0.58) | 4.51 (0.99) | 4.97 (0.16) | 4.66 (0.80) | 4.23 (0.98) | 4.55 (0.77) | 4.91 (0.39) | 4.90 (0.31) | 4.57 (0.82) | 4.23 (1.39) | 4.74 (0.78) | 4.85 (0.37) | 4.44 (1.08) | 4.20 (1.26) | 4.69 (0.89) | 4.92 (0.28) |
| 13. What they want to know about  illness/health condition | 4.00 (1.11) | 3.86 (1.14) | 4.56 (1.00) | 4.42 (1.11) | 4.06 (1.14) | 4.16 (1.00) | 4.52 (0.83) | 4.71 (0.53) | 3.77 (1.06) | 3.56 (1.45) | 4.34 (1.19) | 4.46 (0.86) | 3.83 (1.36) | 3.32 (1.57) | 4.19 (1.28) | 4.36 (1.22) |
| 14. Patients’ personal wishes with  regard to their care | 4.59 (0.60) | 4.14 (1.31) | 4.54 (0.65) | 4.31 (0.96) | 4.27 (0.98) | 4.42 (0.81) | 4.03 (1.09) | 4.60 (0.72) | 4.60 (0.74) | 4.35 (1.16) | 4.40 (0.95) | 4.35 (0.98) | 4.37 (1.09) | 4.29 (1.12) | 4.20 (1.28) | 4.20 (1.23) |
| 15. Help patients take part in  decisions | 4.17 (0.97) | 3.97 (1.40) | 4.27 (0.93) | 4.11 (1.11) | 4.00 (1.03) | 4.37 (0.72) | 3.87 (1.07) | 4.35 (0.84) | 4.49 (0.78) | 4.27 (1.19) | 4.43 (0.74) | 4.27 (0.96) | 4.00 (1.31) | 4.20 (1.08) | 4.11 (1.26) | 4.12 (1.30) |
| 16. Encourage patients to express  their opinions | 4.03 (1.13) | 3.60 (1.38) | 4.51 (0.65) | 4.26 (0.99) | 4.12 (1.14) | 4.45 (0.78) | 4.06 (1.00) | 4.59 (0.63) | 4.37 (0.77) | 4.00 (1.23) | 4.38 (0.99) | 4.28 (1.17) | 3.89 (1.33) | 4.12 (1.05) | 4.20 (1.13) | 4.44 (0.82) |
| 17. Ask patients at what time they  would prefer to wash | 3.66 (1.26) | 2.97 (1.65) | 4.78 (0.42) | 4.42 (1.20) | 2.79 (1.43) | 3.21 (1.60) | 4.52 (1.06) | 4.87 (0.43) | 3.71 (1.36) | 3.15 (1.54) | 4.34 (0.73) | 4.48 (0.82) | 3.31 (1.35) | 2.76 (1.33) | 4.46 (0.92) | 4.42 (1.06) |
