# Supplementary material for: Feasibility and early effectiveness of the Tell‐us Card communication tool to increase in‐hospital patient participation: a cluster randomised controlled pilot study
Source: Scand J Caring Sci. 2020 Sep 21;35(3):911–22. doi: 10.1111/scs.12909 (PMC8451905; doi:10.1111/scs.12909)
Supplement: Supplementary file 2 — Appendix S2. Mean scores per item Quality from the Patients Perspective questionnaire. [file SCS-35-911-s002.docx]

| Appendix S2 Mean scores per item Quality from the Patients Perspective questionnaire | | | | | | | | | |  |
| --- | --- | --- | --- | --- | --- | --- | --- | --- | --- | --- |
| Mean (SD)  Abbreviated question | Cardiology | | | |  | Surgical | | | | |
|  |  | | | |  |  | | | | |
|  | Control | | Intervention | |  | Control | | Intervention | | |
|  | T0 | T1 | T0 | T1 |  | T0 | T1 | T0 | T1 | |
| 1. Physical caring | 3.86 (0.44) | 3.69 (0.69) | 3.46 (0.88) | 3.77 (0.65) |  | 3.88 (0.33) | 3.76 (0.44) | 3.65 (0.80) | 3.95 (0.21) | |
| 1. Medical care | 3.97 (0.18) | 3.83 (0.56) | 3.83 (0.46) | 3.80 (0.61) |  | 3.77 (0.65) | 3.84 (0.37) | 3.76 (0.68) | 3.92 (0.28) | |
| 1. Pain relief | 3.88 (0.33) | 3.78 (0.51) | 3.78 (0.42) | 3.60 (0.94) |  | 3.88 (0.41) | 3.91 (0.29) | 3.62 (0.99) | 3.96 (0.21) | |
| 1. Treatment waiting time | 3.61 (0.79) | 3.82 (0.46) | 3.81 (0.48) | 3.36 (0.95) |  | 3.82 (0.39) | 3.77 (0.43) | 3.76 (0.68) | 3.76 (0.54) | |
| 5a. Information before procedures | 3.74 (0.51) | 3.75 (0.55) | 3.74 (0.51) | 3.68 (0.77) |  | 3.88 (0.41) | 3.65 (0.69) | 3.75 (0.65) | 3.96 (0.20) | |
| 5b. Information after procedures | 3.76 (0.44) | 3.75 (0.50) | 3.73 (0.58) | 3.64 (0.78) |  | 3.85 (0.44) | 3.79 (0.41) | 3.59 (0.76) | 3.84 (0.37) | |
| 5c. Information after procedures | 3.72 (0.65) | 3.35 (0.84) | 3.43 (0.77) | 3.64 (0.76) |  | 3.81 (0.54) | 3.48 (0.67) | 3.55 (0.83) | 3.74 (0.62) | |
| 5d. Responsible persons | 3.76 (0.65) | 3.67 (0.53) | 3.39 (0.83) | 3.36 (1.08) |  | 3.69 (0.63) | 3.58 (0.81) | 3.58 (0.84) | 3.80 (0.65) | |
| 5e. Responsible persons | 3.94 (0.25) | 3.75 (0.65) | 3.50 (0.84) | 3.69 (0.74) |  | 3.86 (0.43) | 3.81 (0.40) | 3.76 (0.55) | 3.68 (0.56) | |
| 1. Participation | 3.66 (0.70) | 3.45 (0.77) | 3.52 (0.71) | 3.64 (0.76) |  | 3.78 (0.49) | 3.71 (0.55) | 3.43 (0.92) | 3.56 (0.58) | |
| 1. Commitment (doctors) | 3.85 (0.57) | 3.81 (0.62) | 3.58 (0.75) | 3.50 (0.79) |  | 3.79 (0.48) | 3.65 (0.49) | 3.69 (0.80) | 3.80 (0.65) | |
| 1. Commitment (nurses) | 3.75 (0.44) | 3.73 (0.61) | 3.70 (0.68) | 3.73 (0.64) |  | 3.94 (0.24) | 3.88 (0.33) | 3.75 (0.77) | 3.80 (0.50) | |
| 1. Empathic and personal (nurses) | 3.69 (0.67) | 3.59 (0.76) | 3.59 (0.78) | 3.60 (0.77) |  | 3.85 (0.36) | 3.63 (0.71) | 3.69 (0.79) | 3.72 (0.54) | |
| 1. Respect (doctors) | 3.91 (0.38) | 3.87 (0.53) | 3.73 (0.76) | 3.73 (0.64) |  | 3.91 (0.28) | 3.88 (0.33) | 3.83 (0.56) | 3.80 (0.71) | |
| 11. Nutrition | 3.76 (0.50) | 3.59 (0.69) | 3.61 (0.75) | 3.61 (0.69) |  | 3.68 (0.73) | 3.88 (0.33) | 3.71 (0.64) | 3.67 (0.58) | |
| 12. Care equipment | 3.55 (0.69) | 3.86 (0.35) | 3.75 (0.55) | 3.69 (0.48) |  | 3.92 (0.28) | 3.94 (0.25) | 3.73 (0.67) | 4.00 (0.00) | |
| 13. Care room characteristics | 3.67 (0.65) | 3.58 (0.72) | 3.45 (0.71) | 3.70 (0.70) |  | 3.89 (0.40) | 3.62 (0.70) | 3.61 (0.69) | 3.64 (0.70) | |
| 14. Secluded environment | 3.43 (0.98) | 3.13 (1.10) | 2.71 (1.10) | 3.06 (0.94) |  | 3.45 (0.83) | 3.33 (0.98) | 3.35 (0.93) | 3.53 (0.92) | |
| 15. Secluded environment | 3.78 (0.42) | 3.46 (0.90) | 3.43 (0.87) | 3.43 (0.84) |  | 3.73 (0.55) | 3.64 (0.63) | 3.80 (0.41) | 3.33 (1.11) | |
| 16. General atmosphere | 3.76 (0.55) | 3.79 (0.58) | 3.85 (0.44) | 3.76 (0.69) |  | 3.74 (0.51) | 3.81 (0.49) | 3.72 (0.61) | 3.75 (0.53) | |
| 17. Family and friends | 3.94 (0.24) | 3.92 (0.49) | 3.91 (0.29) | 3.80 (0.61) |  | 3.97 (0.18) | 4.00 (0.00) | 3.94 (0.34) | 3.92 (0.41) | |
| 18. Routines | 3.57 (0.77) | 3.41 (0.76) | 3.13 (0.94) | 3.63 ( 0.74) |  | 3.57 (0.63) | 3.28 (0.89) | 3.52 (0.80) | 3.46 (0.72) | |
